# Addressing Bias, Privacy, Security, and Patient Autonomy in Artificial Intelligence (AI)-Driven Healthcare: A Review of Current Guidelines

**DOI:** 10.7759/cureus.103999

**Published:** 2026-02-20

**Authors:** Shruti Singh, Prashant K Singh, Rajesh Kumar, Riya Vaidyar

**Affiliations:** 1 Department of Pharmacology, All India Institute of Medical Sciences, Patna, IND; 2 Department of General Surgery, All India Institute of Medical Sciences, Patna, IND

**Keywords:** algorithmic bias, artificial intelligence (ai), data privacy, digital information security in healthcare act, hipaa and gdpr ai compliance

## Abstract

Integrating artificial intelligence (AI) in healthcare has revolutionized patient care, diagnostics, and operational efficiency. However, the reliance of AI systems on vast amounts of personal data raises significant concerns regarding data privacy, security, and ethical governance. This narrative review examines global regulations, including the General Data Protection Regulation, the Health Insurance Portability and Accountability Act, and Organization for Economic Co-operation and Development guidelines, and contrasts them with India’s evolving data privacy landscape, particularly under the Digital Personal Data Protection Act, 2023. The review explores key ethical challenges, including AI bias, patient consent, data security, and algorithmic transparency, and provides case studies from around the world. The paper concludes with policy recommendations to harmonize international standards, strengthen AI governance in healthcare, and foster ethical AI development.

## Introduction and background

Artificial intelligence (AI) in healthcare refers to the use of intelligent, data-driven algorithms and technologies for improving medical services [1]. In routine practice, AI already supports image interpretation (e.g., radiology triage and diabetic retinopathy screening), clinical risk prediction (e.g., sepsis alerts), and workflow optimization (e.g., summarization of electronic health records and appointment prioritization). These use-cases depend on large-scale personal health data and, therefore, amplify privacy, consent, and accountability concerns.

As AI systems rely on large volumes of patient data to learn and function, data privacy has become critically important, considering that improper use or disclosure can harm patients’ rights and trust. Ensuring strong privacy protections in AI-driven healthcare is, therefore, essential to maintaining patient confidentiality, complying with ethical standards, and upholding public trust in medical AI innovations​ [2,3].

Globally, several major regulations establish frameworks for the privacy of health data. The European Union’s General Data Protection Regulation (EU GDPR) (2018) and the Health Insurance Portability and Accountability Act (HIPAA) (1996) of the USA set national standards to protect medical records and personal health information, prohibiting disclosure without patient consent or knowledge in most cases​ [2,3]. The OECD’s privacy guidelines and AI principles advocate for the responsible use of data and “trustworthy AI that respects human rights and democratic values”​ [4].

Despite the availability of major legal instruments and ethics frameworks, there remains a persistent implementation gap: clinicians, administrators, and developers often lack consolidated guidance on 1) how specific privacy obligations translate into AI system design and deployment, and 2) how to operationalize patient autonomy, transparency, and bias mitigation across the AI lifecycle. This gap is particularly relevant in India, where sector-wide digital health expansion is progressing faster than healthcare-specific compliance capacity.

This lack of awareness can lead to numerous patient-related issues, including breaches of privacy, ethical concerns, and potential legal repercussions. This necessitates a consolidation of key issues, principles, and implications of the use of AI in healthcare, which led to the development of this review to ensure widespread awareness and informed implementation.

Accordingly, this review 1) synthesizes international privacy and AI governance frameworks relevant to healthcare, 2) contextualizes India’s evolving legal landscape (IT Act/Sensitive Personal Data or Information (SPDI) rules to Digital Personal Data Protection (DPDP) Act, and Ayushman Bharat Digital Mission (ABDM)), 3) summarizes recurring ethical and compliance risks with real-world case illustrations, and 4) proposes actionable policy and institutional recommendations for ethical AI implementation.

## Review

Methodology

This narrative review followed a structured but nonsystematic approach. Although Preferred Reporting Items for Systematic reviews and Meta-Analyses reporting is intended for systematic reviews, we provide a brief transparency summary: databases searched, date last searched, broad inclusion criteria, and thematic synthesis approach. No quantitative synthesis (e.g., meta-analysis or meta-regression) was planned or performed because the included sources comprised legal instruments, ethics frameworks, and heterogeneous empirical designs not suitable for pooled estimation.

Searches were conducted in PubMed and Google Scholar, supplemented by official government and regulatory databases for primary legal texts and policy guidance (e.g., EU, US, OECD, WHO, and India). Search terms included combinations of AI, healthcare, medical data, privacy, data protection, consent, governance, bias, explainable AI, federated learning, differential privacy, GDPR, HIPAA, and DPDP Act. The literature search covered publications from January 2020 to January 2026, with specific attention to post-2022 guidance on generative AI and large language models in healthcare.

Eligibility criteria included English-language sources comprising the following: 1) primary legal and regulatory instruments; 2) ethics frameworks and standards; 3) peer-reviewed empirical studies or reviews addressing privacy, security, or ethics of AI in healthcare; and 4) high-impact case reports or investigations directly relevant to healthcare AI data use. Exclusion criteria were as follows: sources unrelated to healthcare AI privacy, commentary lacking traceable primary support when higher quality evidence was available, and duplicate reporting of the same case without additional substantive information.

Sources were screened by title and abstract for relevance, followed by full-text review. Evidence was synthesized thematically across four domains: regulatory frameworks, the Indian legal landscape, ethical and compliance risks, and future governance recommendations. Because the objective was interpretive and policy-oriented, formal risk-of-bias appraisal was not performed, which is consistent with narrative review methodology. Where evidence was heterogeneous or contested, consensus guidance and landmark empirical studies were prioritized, and conclusions were framed accordingly.

The authors report using an AI tool based on a large language model (ChatGPT, OpenAI) for grammatical editing and stylistic refinement only. The tool did not generate scientific content, interpret data, or influence analytical or conceptual judgments. All authors independently reviewed and approved the final manuscript and accept full responsibility for the accuracy, integrity, and quality of the work.

Global consensus on AI and data privacy in healthcare

International Regulations and Frameworks

Around the world, governments and standards bodies have recognized the need to regulate data privacy in healthcare, especially as AI becomes more prevalent. Europe’s GDPR is a global benchmark, classifying health data as “sensitive personal data” with strict protections. It mandates a lawful basis (e.g., explicit patient consent) for processing and grants individuals rights over their data. Its principles, consent, transparency, data minimization, and accountability, have influenced privacy laws worldwide [5]. In the US, HIPAA regulates the handling of protected health information (PHI) by healthcare providers, insurers, and associates. It ensures confidentiality, security, and restricted use of PHI, requiring authorization or legal justification (e.g., for treatment or payment). Though predating AI, HIPAA applies to AI tools in healthcare, ensuring patient data remain protected [6]. The OECD Privacy Guidelines (1980, updated 2013) established key principles such as data limitation, security, and accountability, which influenced national laws. The OECD AI Principles (2019) set the first intergovernmental AI standards, promoting trustworthy AI that upholds human rights, transparency, fairness, and accountability, especially in the handling of health data [4,7]. The WHO's AI ethics principles protect human autonomy, ensuring that patients control their healthcare decisions and data with privacy and informed consent safeguards. Other key principles include promoting well-being, transparency, accountability, inclusiveness, and sustainable AI in healthcare (Table 1) [8,9].

**Table 1:** Comparison of international AI healthcare data privacy laws A comparison of key global data privacy laws/frameworks was done as they relate to AI in healthcare. GDPR and HIPAA are legally enforceable regulations in the EU and the US, respectively, while the OECD guidelines are soft law influencing many jurisdictions [4-9] AI: artificial intelligence; GDPR: General Data Protection Regulation; EU: European Union; HIPAA: Health Insurance Portability and Accountability Act; PHI: protected health information; OECD: Organization for Economic Co-operation and Development; LMM: large multimodal models

Law/framework (region)	Scope and application	Key provisions for health data and AI
GDPR (EU, 2018)	Broad data protection law covering all personal data in EU; applies to health data as “special category” sensitive data [5]	Requires a lawful basis (e.g., explicit consent) to process health data
		Enshrines data subject rights (access, rectification, erasure, objection), including right not to be subject to solely automated decisions with legal effects (Article 22)
		Imposes data protection by design/default and high penalties for breaches. GDPR’s principles (transparency, purpose limitation, data minimization, etc.) strongly influence AI data use
HIPAA (USA, 1996)	Sectoral law for healthcare entities (providers, insurers, clearinghouses) and their business associates handling PHI [6]	Requires safeguarding of PHI and restricts uses/disclosures without patient authorization, except for treatment, payment, and operations
		Includes security rule mandating administrative, physical, and technical safeguards for e-PHI (relevant for health IT and AI systems)
		Allows data sharing via Business Associate Agreements, under which AI vendors can receive data for certain purposes (must remain within HIPAA bounds). No specific provisions on AI, but general privacy and security rules apply to any AI handling PHI
OECD Privacy Guidelines (Global, 2013)	Nonbinding international framework adopted by OECD countries (and influential globally) [4,7]	Sets out 8 core principles: Collection Limitation, Data Quality, Purpose Specification, Use Limitation, Security Safeguards, Openness, Individual Participation, Accountability
		Though general, these principles guide health data handling: e.g., collect only necessary health data, use it only for specified purposes (like a particular AI tool) unless consent for new use is obtained, ensure strong security, and be accountable for compliance
		Influenced laws like GDPR reinforce the need for transparency and consent in AI-driven health data use
WHO	Nonbinding global ethical guidance on AI in health, focused on public health, clinical care, and research; intended for governments, developers, and health providers [8,9]	Establishes six core ethical principles: protect human autonomy, promote human well-being and safety, ensure transparency and explainability, foster responsibility and accountability, safeguard inclusiveness and equity, and promote responsive and sustainable AI in healthcare
		Calls for ethics- and privacy-by-design, human oversight, informed consent, and protection of clinician-patient relationships
		Provides extensive governance recommendations (40+ points) for LMMs, covering data privacy, bias prevention, cybersecurity, clinical safety, and environmental sustainability
		Urges documentation of data practices: clearly define roles of data controllers/processors, legal bases, types of data, and collection methods, promoting transparency and accountability
		Highlights the need for national regulation, public monitoring, and multistakeholder accountability mechanisms to ensure AI benefits do not exacerbate health inequities

These international guidelines, while not legally binding, reflect common themes that have emerged: the need for informed patient consent, strong data security measures, transparency in how AI systems use data, and mechanisms to prevent bias or discrimination.

Data privacy and security are crucial for trustworthy AI in healthcare, as data breaches are more frequent in this sector than in any other. Strong security controls (encryption, access restrictions, and network safeguards) are essential to protect sensitive health information from cyber threats. Regulators emphasize that privacy requires security, urging healthcare AI vendors to secure data, vet third-party tools, and prevent cyberattacks, with high-profile breaches driving stricter enforcement [3,9].

Preventing AI bias and discrimination in healthcare is a key concern, as biased training data can lead to inequitable care. For example, a widely used clinical risk algorithm underestimated the needs of Black patients due to biased data. Regulators stress fairness, inclusiveness, and equity, requiring AI developers to test for bias, use diverse datasets, and adjust algorithms to prevent disparities. Just as human clinicians must provide equitable care, AI tools must also meet fairness standards to comply with emerging regulations [9,10].

Patient autonomy and consent are central to data privacy laws like GDPR and HIPAA, granting individuals control over their health data. AI development using patient records typically requires informed consent, unless exempted. Transparency is key: patients must be informed if their data train AI or influence decisions about their care. Lack of consent can spark a backlash, as seen in Google DeepMind’s National Health Service (NHS) case. GDPR even allows patients to opt out of automated decisions [11,12].

Case Studies of AI-Related Data Privacy Issues

To illustrate these themes, several notable cases around the world have highlighted the privacy challenges of AI in healthcare and its legal implications.

Google DeepMind and the UK’s NHS (2015-2017): In a notable case, the Royal Free London NHS Trust provided Google’s DeepMind with 1.6 million patient records without informing patients. The data were used to develop the AI-powered Streams app for detecting acute kidney injury. In 2017, the UK’s Information Commissioner’s Office ruled that the hospital had breached the Data Protection Act due to inadequate transparency (lack of informed consent) and the lack of a legal basis for data transfer. While no fines were issued, it led to new NHS guidelines on technology partnerships and reinforced the importance of privacy compliance to maintain public trust and avoid legal challenges [11].

Google and Ascension’s “Project Nightingale” (2019): In the US, Google’s partnership with Ascension under “Project Nightingale” involved transferring over 50 million patient records to Google for AI-driven healthcare improvements. The project remained secret until media reports in November 2019, sparking public outcry over patient data being shared without consent. Google and Ascension defended the initiative as legal under HIPAA, citing a Business Associate Agreement (BAA) that allowed data sharing for healthcare operations without patient consent. However, critics argued that this exploited a legal loophole, the lack of patient notification, and that BAAs designed in the early 2000s might be too “vague and out of date” for modern big data uses. The US Department of Health and Human Services investigated but found no HIPAA violations. Despite legal compliance, the controversy exposed a gap between regulations and public expectations, fuelling calls for greater transparency in health data use and potential updates to privacy laws. It underscored the importance of maintaining patient trust in AI-driven healthcare initiatives [13].

Other examples: Several incidents continue to shape global AI privacy debates. In 2021, Roche’s subsidiary, Flatiron Health, faced scrutiny in Europe for training AI on real-world oncology data without explicit patient consent, raising GDPR concerns. In the UK, a telehealth AI system accidentally exposed patient consultation videos due to a software bug, highlighting privacy risks in AI-driven telemedicine. Additionally, studies have shown AI’s ability to reidentify individuals from anonymized data: one 2018 study found an algorithm could correctly reidentify ~85% of adults in a health survey by linking it with other datasets. These cases underscore the ongoing challenges in balancing AI innovation with data privacy and security​ [14-17]. This technical finding challenges assumptions in privacy regulations about deidentification being sufficient protection, and regulators now warn that robust anonymization or newer techniques (like differential privacy) may be needed when sharing data for AI research. The global trend is toward strengthening guidance and oversight so that innovative AI applications do not come at the expense of patient privacy or ethical standards.

Indian Perspective

Evolving data privacy regulations in India: Historically, India lacked a dedicated health data privacy statute. The primary legal safeguard was the Information Technology Act, 2000, and its associated rules. Specifically, the IT Act’s 2011 “Reasonable Security Practices and Procedures and SPDI" Rules identified health information as sensitive personal data and required entities to implement adequate security measures to protect it and obtain consent for disclosure. However, enforcement was relatively weak and limited to the private sector handling data: government hospitals and programs were not directly covered by Section 43A, leaving gaps in protection​ [18-20].

Recognizing the need for stronger safeguards, India’s Ministry of Health proposed the Digital Information Security in Healthcare Act (DISHA) in 2018. DISHA was a draft bill aimed at specifically regulating digital health data. Its goal was to give patients greater control over their health data and to standardize how data could be collected, stored, and shared in the healthcare sector. DISHA sought to establish a National eHealth Authority (NeHA) and corresponding state authorities to oversee health data exchanges​ [21-23]. It mandated that patients’ informed consent be required for any use of their identifiable health information and outlined penalties for breaches. In essence, DISHA was India’s attempt at a HIPAA-like health privacy law, tailored to the Indian context. Notably, it intended to make the “data owner” (the patient) the ultimate authority over who accesses their digital health records. However, DISHA never made it to a final vote. In 2019, as India was also developing a general data protection law, the health ministry decided to subsume DISHA’s provisions into the broader personal data protection framework being drafted by the central government​ to avoid regulatory overlap by having one comprehensive law cover all personal data, including health, rather than separate sectoral laws [21-23].

After several iterations and debates, India finally passed the DPDP Act, 2023 (DPDP Act), a landmark general privacy law that impacts all sectors, including healthcare. The DPDP Act (introduced as a bill in 2022 and passed in August 2023) applies to all “digital personal data” and sets out rules for how such data may be collected and processed​ [24]. The DPDP Act mandates consent for processing personal data, with exceptions for medical emergencies, public health, and government functions. It holds "Data Fiduciaries" accountable for accuracy, security, and timely deletion; introduces penalties up to ₹250 crores for violations; permits most cross-border data transfers; and establishes the Data Protection Board of India to enforce compliance, marking a significant step in India's healthcare data privacy landscape (Table 2) [25-29].

**Table 2:** Key provisions of the DPDP Act (India) and practical implications in healthcare and AI The DPDP Act, 2023, regulates digital personal data in India, emphasizing privacy, consent, and accountability. It applies to healthcare institutions, AI developers, telemedicine platforms, and medical research entities handling patient data. Compliance mandates explicit consent, data security, bias audits for AI models, and transparency in automated decisions. The DPBI oversees enforcement, with penalties up to ₹250 crore for violations. AI-driven healthcare and cross-border data collaborations must align with DPDP Act standards to ensure privacy, security, and fairness [21,25-29] AI: artificial intelligence; DPDP: Digital Personal Data Protection; SDF: significant data fiduciary; DPO: Data Protection Officer; RTBF: right to be forgotten; DPBI: Data Protection Board of India

Key provision	Description	Practical implications in healthcare and AI
Applicability and scope	The DPDP Act applies to personal digital data collected online or digitized from offline sources. Covers processing by Indian entities and foreign entities targeting Indian data subjects [21,25]	Any AI-driven health analytics or diagnosis tool that processes Indian patient data (even if located abroad) must comply
		Researchers using digitized patient records must ensure compliance
Consent-based data processing	Data processing is only allowed with explicit consent, except in certain cases (e.g., emergencies, government functions). Consent must be clear, informed, and revocable [21,25]	Hospitals and clinics must obtain explicit patient consent before using AI-based diagnostics or EHRs
		Companies cannot use patient health data to train AI models without explicit patient approval
		Dynamic consent models are needed for longitudinal research studies using patient data over time
Legitimate use exceptions	Certain uses are permitted without explicit consent under the DPDP Act, including processing for medical emergencies (life-saving care and disease prevention), public health initiatives such as epidemic surveillance, and law-enforcement requirements [21,25,28,29]	AI triage systems in ERs can use patient data without consent for critical care
		Government AI tools tracking disease outbreaks can collect anonymous health data
Data fiduciary responsibilities	Any entity processing personal data is a data fiduciary and must ensure lawful processing, accuracy, security, and deletion after the intended use [21,24,25]	Hospitals and healthcare providers need robust data management policies to avoid liability
		AI developers and research institutions must ensure data minimization and fair AI model training practices
SDF designation	The DPDP Act classifies hospitals and AI healthcare platforms handling sensitive personal data as SDFs, mandating appointment of a Data Protection Officer, periodic audits, and risk assessments to ensure compliance with enhanced accountability requirements under Sections 10-11 of the Act [21,28,29]	Large hospitals and telemedicine platforms need to hire a DPO
		AI start-ups handling large-scale patient data must implement regular audits and compliance checks
Children’s data protection	Processing of children’s personal data (under 18) requires verifiable parental consent and prohibits behavioral profiling [24-26]	AI-powered pediatric tools must obtain guardian consent for child patients before using predictive health analytics
		EdTech and Health Monitoring Apps for minors cannot use targeted AI ads or personalized recommendations for minors
Data storage and localization	The government may restrict cross-border transfers of sensitive personal data to noncompliant countries [27-29]	Companies hosting AI-driven health analytics tools on foreign servers/cloud-based AI models may need local storage solutions
		International data-sharing agreements needed for multicenter trials
Right to data access and correction	Individuals can request access to their data, corrections, or deletions from data fiduciaries [24-26]	Patients can demand corrections in EHR systems and AI-driven patient records
		If an AI incorrectly classifies a patient’s risk based on a predictive AI model in Health Insurance, they can request a review
RTBF	Data subjects can request deletion of their personal data after the intended purpose is fulfilled [23-25]	Hospitals and labs must delete patient records once retention periods end
		AI Models trained on personal data may need to remove patient-specific data upon request
Data breach reporting	Mandatory reporting of data breaches to the Data Protection Board and affected individuals [26,27,29]	Hospitals and EHR providers must immediately report breaches (e.g., cyberattacks leaking patient data)
		AI companies handling health data need incident response plans to manage privacy violations
Data processing for research and public good	Allows data processing for research, statistical analysis, and public welfare under government-approved guidelines [24,25]	AI research in genomics can continue under defined ethical safeguards
		AI models tracking chronic disease patterns can process anonymized patient data and conduct other public health studies
DPBI	Regulatory authority overseeing compliance, investigating violations, and imposing penalties [29]	Ensures fair AI decision-making in healthcare
		Can impose fines of up to ₹250 crore ($30M) for major breaches
		Telemedicine Platforms risk fines for data misuse if patient consent isn’t properly obtained

In addition to the DPDP Act, India's ABDM issues unique Health IDs (Ayushman Bharat Health Account numbers) and enables consent-based digital health record sharing across hospitals. While not a law, ABDM aligns with the DPDP Act's goal of secure, patient-controlled data exchange, with its success relying on strong privacy enforcement [30].

Challenges in Implementation and Enforcement

Translating India’s new data protection rules into practice, especially in the healthcare sector, faces several challenges. First, the healthcare system in India is vast and fragmented, ranging from large corporate hospital chains to small clinics and labs, and awareness of data privacy obligations is uneven. Without strong legal safeguards, many healthcare providers in India historically shared or sold patient data to third parties without consent. For example, an investigation in 2022 found clinics openly selling pregnant women’s ultrasound data to stem cell banks looking for potential customers, enabled by a lack of regulation and low enforcement. With the DPDP Act, such practices become illegal, but ensuring compliance among thousands of small health providers is a massive enforcement challenge. Many mid-size hospitals do not even have proper electronic record systems or audit logs to track who accessed data, making it hard to detect unauthorized use. The government will need to invest in educating healthcare entities about the new law, possibly starting with larger institutions setting examples of compliance [31].

Another challenge is the absence of a sector-specific regulator for health data. While the Data Protection Board can handle complaints and breaches across all domains, healthcare might benefit from a dedicated oversight body (as DISHA had envisioned with NeHA). Without specialized regulators or clear guidelines tailored to healthcare AI, there may be ambiguity in how to apply certain provisions of the DPDP Act. For instance, the law provides exceptions that could be interpreted broadly in medical contexts, such as processing without consent for “public interest” or “research” (Table 2). It remains to be seen how these will be balanced against patient consent rights. Privacy advocates in India have voiced concern that the government has wide powers to exempt agencies or projects from the law, which could undermine enforcement​ [32]. Notably, the DPDP Act allows the central government to exempt certain data processing by government agencies from almost all provisions. A former Supreme Court Justice, B.N. Srikrishna (who chaired the committee that drafted the earlier 2018 bill), criticized this, warning that giving unchecked exemption powers could be “an invitation to arbitrariness”​ [32]. In healthcare, if too many programs (such as national health registries or health research schemes) receive exemptions, it might weaken protections for citizens. Therefore, a key challenge is ensuring that privacy rules are uniformly applied and that any exemptions are narrow and justified.

India also faces the task of building infrastructure and standards to support privacy-compliant AI in healthcare. As noted in one analysis, the current legal landscape is fragmented, and there is limited legal scholarship or guidance on digital health​ [19]. This fragmentation creates uncertainty among healthcare providers about how to implement concepts such as informed consent for AI, data anonymization standards, and data-sharing agreements. The country will need updated clinical research guidelines, hospital policies, and possibly an extension of existing healthcare quality standards to include data protection. Additionally, enforcement capacity is a concern: the Data Protection Board will need technical experts to audit AI algorithms and data flows in healthcare, which is a new frontier. The Indian IT Ministry and Health Ministry may have to coordinate closely (perhaps even revive some of the DISHA draft’s specific rules) to create sectoral rules under the DPDP Act for health data.

Case Studies: India’s AI and Healthcare Data Landscape

While India’s adoption of AI in healthcare is still emerging, there have already been instances highlighting data privacy issues.

AIIMS cyberattack (2022): In November 2022, the All India Institute of Medical Sciences (AIIMS) in Delhi faced a major ransomware attack, crippling hospital systems and exposing 40 million patient records. The breach highlighted India’s cybersecurity gaps, as the DPDP Act was not yet in place, leaving unclear breach response protocols. With rising cyberattacks on digitized health data, the incident underscored the urgent need for stronger data protection laws and cybersecurity in healthcare to maintain public trust [33-36].

Rampant data sharing for AI/analytics: Investigations in India have exposed unauthorized health data sharing, often under the guise of AI research or business deals. A diagnostic lab chain shared insufficiently anonymized patient data with a health-tech startup, raising privacy concerns. During COVID-19, the Aarogya Setu app (Informatics Center, Ministry of Electronics and Information Technology, New Delhi) collected massive personal health data, sparking fears of misuse due to a lack of a privacy law. Advocates stressed that data given for pandemic control should not be repurposed without explicit consent. These concerns influenced the DPDP Act, reinforcing strict purpose limitations and consent rules [37].

Emerging AI health startups and data use: India’s AI-driven health startups, such as SigTuple (blood slide analysis) and Niramai (breast cancer screening), collaborate with hospitals for training data. While no breaches have been reported, data sharing remains a gray area under the new DPDP Act. If hospital-shared scans are truly anonymized, they fall outside regulation, but full anonymization is challenging. A cautious approach suggests treating deidentified data as sensitive, requiring patient consent or ethics approval. Some hospitals now include broad consent in admission forms, but its adequacy under the new law is uncertain. Regulatory guidelines may be needed to balance innovation and patient privacy [38,39].

Ethical and compliance challenges in AI-driven healthcare

Implementing AI in healthcare brings not only opportunities but also a host of ethical and compliance challenges. Ensuring that AI systems are used in a manner that is fair, safe, and lawful is a complex task (Table 3).

**Table 3:** Ethical risks in AI-driven healthcare and mitigation strategies Common ethical and compliance issues are encountered with AI in healthcare, along with strategies to mitigate these risks. Proactive measures, from technical solutions (encryption, federated learning) to policy interventions (consent management, transparency requirements), can help ensure AI systems are used in a manner that is fair, secure, and respectful of patient rights [3,9-12,21-23,29,40-55] AI: artificial intelligence; NIST: National Institute of Standards and Technology; HIPAA: Health Insurance Portability and Accountability Act; DISHA: Digital Information Security in Healthcare Act

Ethical/compliance issue	Description of risk	Mitigation strategies
AI bias and fairness	AI algorithms may perpetuate bias or discrimination present in historical health data, leading to unequal care (e.g., underdiagnosis in minorities or women). This can result in ethically unfair outcomes and potential legal liability under antidiscrimination laws	Use diverse, representative datasets for training [9]
		Regularly evaluate model outputs for disparate impact across demographic groups [10,11]
		Implement bias correction techniques and algorithmic audits throughout the AI lifecycle [9]
		Follow guidelines (e.g., WHO’s equity principle, NIST AI framework) to ensure AI tools are tested for fairness and do not exacerbate health disparities [40]
Data privacy and reidentification	Large-scale data use in AI raises privacy risks; even deidentified datasets can be reidentified by savvy algorithms or linked to other data. Patients’ personal health information could be exposed without their knowledge	Apply privacy-by-design: minimize data collection and retention to what's necessary for the AI’s purpose [41-45]
		Utilize privacy-preserving techniques like federated learning (train AI models without centralizing data) and differential privacy (add noise to data or queries to protect individual identities) [46-49]
		Enforce strict data use agreements: data obtained for one project cannot be used for other purposes without fresh consent (prevent “function creep”) [46-49]
Data security breaches	Healthcare data are a prime target for cyberattacks; AI systems aggregating data may introduce new vulnerabilities. A breach can expose sensitive patient info, violating privacy and damaging trust	Implement robust cybersecurity measures: encryption of data at rest and in transit, multifactor access controls, and continuous network monitoring [3,50-52]
		Conduct regular security audits and penetration testing, including for third-party AI vendors (ensure business associates follow equivalent security standards) [3,50-52]
		Maintain an incident response plan in line with regulations (e.g., HIPAA Breach Notification Rule) to quickly contain and report any data breaches, mitigating impact [3,52]
Lack of transparency (explainability)	Many AI models (e.g., deep learning) are “black boxes,” making it hard for clinicians and patients to understand the rationale behind an AI’s decision or prediction. This opaqueness can reduce trust and complicate accountability	Prefer or develop explainable AI methods in high-stakes uses: models that can provide human-interpretable reasons or highlight relevant patient factors for a given recommendation [12]
		Ensure transparency about AI system design and limitations: publish information about the algorithm’s performance, training data, and intended use [53]
		Follow emerging standards or regulations on AI transparency (such as documentation and algorithmic impact assessments) so that there is a clear record for auditing and for users to understand how the AI influences care decisions [12]
Informed consent and data ownership	Patients may be unaware that their health data are used to train AI, or that AI is involved in their care decisions. Traditional consent processes might not cover these novel uses, raising ethical issues. Additionally, patients have little say in or benefit from the secondary use of their data	Update informed consent practices: explicitly inform patients if their data will be used for AI development or if an AI tool will assist in their care, including relevant risks/benefits [54,55]
		Where feasible, implement dynamic consent models that allow patients to choose how their data can be used (e.g., some may consent to research/AI use, others may opt out) [54,55]
		Establish clear data governance policies that treat patients as key stakeholders, for instance, involve patient representatives in AI project oversight, and consider frameworks that grant patients more control or share aggregate benefits (while still protecting privacy) [21-23]
		In India, for example, the proposed DISHA law aimed to grant patients greater rights over their digital health data [29]

AI Bias and Fairness

A major ethical challenge in healthcare AI is algorithmic bias, which can produce unequal or unjust clinical outcomes. Because AI systems learn from historical datasets, they may inherit and amplify existing social and healthcare disparities. For example, a widely used clinical risk algorithm was shown to underestimate illness severity in Black patients compared with White patients with similar health status [10,11]. Such bias is ethically unacceptable and may also create legal exposure under antidiscrimination frameworks. Mitigating bias requires diverse and representative training datasets, systematic bias detection and correction, and regular audits of performance across demographic groups [9]. Emerging governance frameworks, including the National Institute of Standards and Technology AI Risk Management Framework and the proposed EU AI Act, emphasize fairness monitoring for high-risk medical AI [40]. Interdisciplinary oversight involving clinicians and ethicists further supports equitable and clinically appropriate AI deployment.

Data Privacy and Reidentification

AI-driven healthcare relies on large datasets, raising substantial privacy risks even when data are deidentified. Advanced analytical techniques can sometimes reidentify individuals by linking anonymized health data with external datasets, challenging the assumption that deidentification alone ensures privacy [41,42]. Under frameworks such as GDPR, reidentified data are again treated as personal data and subject to full legal protections. In addition, AI models themselves may leak information through model inversion or membership inference attacks. To mitigate these risks, experts advocate privacy-enhancing approaches such as differential privacy and federated learning, which enable model training while minimizing exposure of individual-level data [43-45]. Regulators increasingly promote a privacy-by-design approach, emphasizing data minimization, strong anonymization practices, and strict purpose limitation to prevent unauthorized secondary use of patient data [46-49].

Data Security and Breach Risk

In addition to privacy concerns, data security is a major challenge in AI-driven healthcare because medical data are highly valuable, and healthcare systems are frequent targets for cyberattacks. AI integration can increase system vulnerability by linking multiple platforms and aggregating large datasets, creating additional entry points for unauthorized access. Major breaches, such as the Anthem incident in the United States and the AIIMS cyberattack in India, illustrate the scale of potential harm to patients and institutions [34,50,51]. Effective protection requires end-to-end security of training databases, AI outputs, and connected clinical devices, as well as safeguards against adversarial manipulation of models. Regulatory standards such as the HIPAA Security Rule mandate administrative, physical, and technical protections, including encryption, strict access controls, and continuous monitoring, and similar scrutiny is increasingly expected of third-party AI vendors. Ongoing risk assessments, security audits, and robust incident response plans are now viewed as essential components of ethical AI governance in healthcare [3,52].

Transparency, Explainability, and Accountability

The opacity of many AI systems, particularly deep learning models, poses challenges for trust and accountability in healthcare. When clinicians and patients cannot understand how an AI system generates recommendations, confidence in its use may decline, and responsibility for errors becomes difficult to assign. Ethical frameworks, including WHO guidance, emphasize the need for transparency and explainability in health AI [12]. While full algorithmic disclosure may not always be feasible, meaningful transparency can be achieved by reporting training data sources, performance metrics, and known limitations. Explainable AI techniques that highlight influential patient factors are increasingly used to support clinical interpretation [53]. From a compliance perspective, thorough documentation, validation, and clear assignment of human oversight are essential. AI tools should function as decision-support systems under clinician responsibility, with regulatory review processes (e.g., Food and Drug Administration (FDA) oversight) helping establish safety standards. Ethical accountability further requires mechanisms for patient review and redress when AI-assisted decisions cause harm [12].

Informed Consent and Data Ownership

In clinical practice, patients often consent to treatment and routine data use without being explicitly informed that their data may contribute to AI development. Meaningful informed consent in AI-driven healthcare, therefore, requires clear disclosure of secondary uses while avoiding information overload. Current debates around data ownership and benefit-sharing highlight a shift toward more patient-centered governance. Frameworks such as India’s proposed DISHA and the DPDP Act emphasize explicit consent and purpose limitation, reinforcing patient control over digital health data [21-24]. Similarly, GDPR requires a clear legal basis for AI-related data processing, typically consent or tightly regulated research exceptions [27]. Ethical discussions increasingly support dynamic consent models that allow patients to adjust their participation over time and encourage patient involvement in data governance through mechanisms such as advisory boards or data trusts [54]. Effective consent frameworks must balance respect for autonomy with the practical needs of research, as transparency and trust are critical to sustaining public willingness to share health data [55].

Future directions and policy recommendations

**Figure 1:**
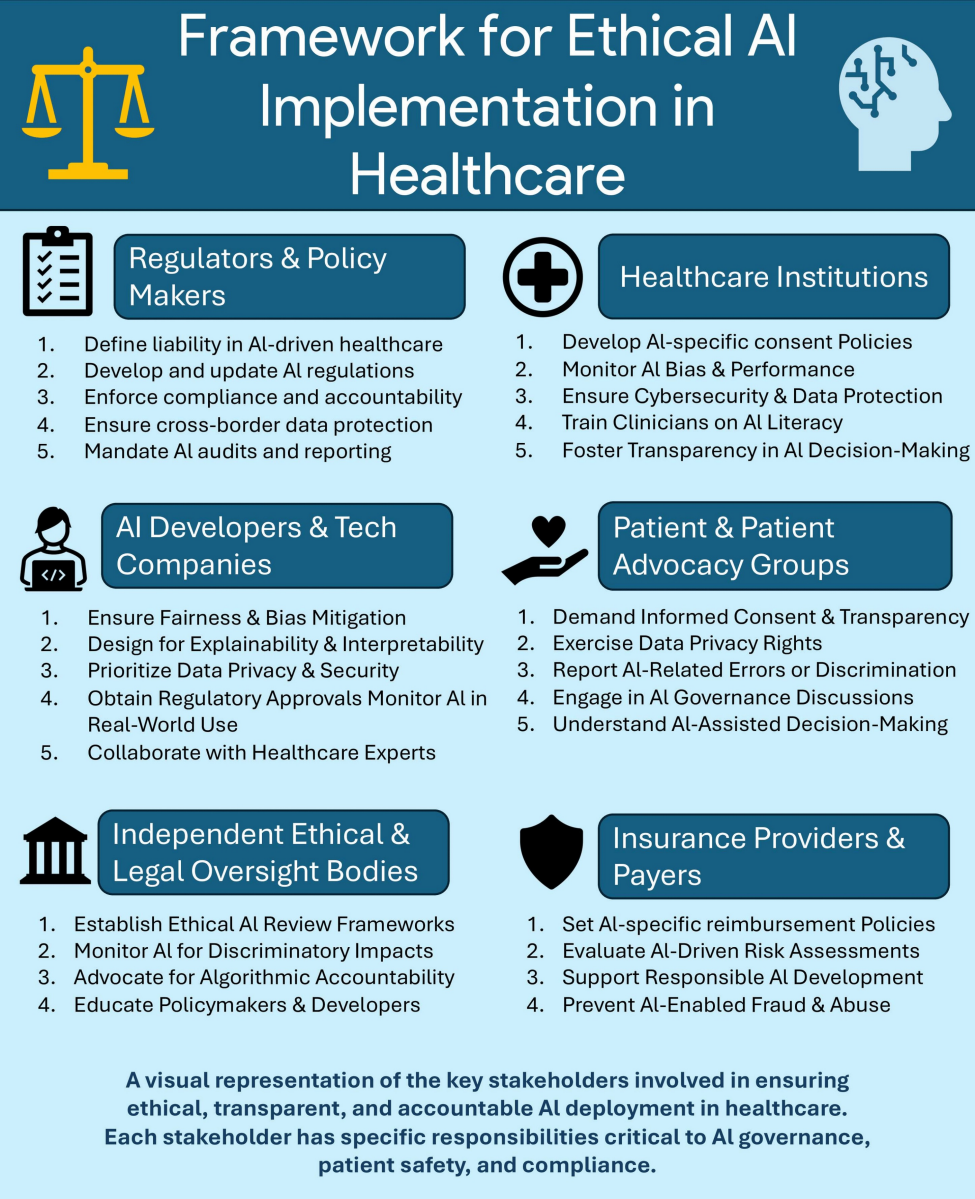
Framework for ethical AI implementation A multistakeholder governance framework essential for ethical, transparent, and accountable AI deployment in healthcare is illustrated. It delineates the roles and responsibilities of six key stakeholder groups: regulators and policymakers, healthcare institutions, AI developers and tech companies, patient advocacy groups, independent ethical and legal oversight bodies, and insurance providers. Responsibilities include ensuring AI-specific consent, preventing algorithmic bias, maintaining data privacy, establishing liability and audit mechanisms, fostering transparency, and protecting patient rights. The model aligns with global best practices and Indian regulatory provisions under the Digital Personal Data Protection Act, 2023, and emerging AI ethics frameworks [9,13,14,18,20,21] Image credit: This is an original image created by the author Shruti Singh

As AI continues to transform healthcare, robust governance frameworks are essential to balance innovation with ethical and privacy safeguards. Key recommendations include strengthening AI governance, such as establishing regulatory oversight mechanisms akin to clinical trial approvals and enforcing algorithmic accountability through audits and compliance checks [12,56-60]. Enhancing security and ethical AI development is crucial, with a focus on privacy-preserving techniques (e.g., federated learning and homomorphic encryption) and interdisciplinary design to mitigate biases [2,61-66]. Global and national standards alignment will facilitate AI deployment across borders, necessitating international certification systems and harmonized data privacy regulations to support multicenter research [67-77]. Education and ethical culture development should integrate AI ethics into medical training, ensuring healthcare professionals and AI developers recognize ethical concerns and biases in AI applications [78-83]. Continuous policy iteration and research are necessary to adapt governance models to emerging AI technologies, funding research on AI ethics, and incentivizing compliance with privacy and fairness standards (Table 4, Figure 1) [84-94].

**Table 4:** Future directions and policy recommendations for AI in healthcare This table presents strategic recommendations and corresponding action points for ethical, secure, and globally harmonized AI governance in healthcare. It aligns proposed actions with existing national and international initiatives across domains such as regulation, cybersecurity, education, and stakeholder engagement to ensure responsible AI adoption and implementation [2,12,22,51,57-94] AI: artificial intelligence; EU: European Union; FDA: Food and Drug Administration; SaMD: Software as a Medical Device; IRB: institutional review board; AiaMD: AI as a medical device; OECD: Organization for Economic Co-operation and Development; DPDP: Digital Personal Data Protection; HIPAA: Health Insurance Portability and Accountability Act; EU GDPR: European Union’s General Data Protection Regulation; CME: continuing medical education; DISHA: Digital Information Security in Healthcare Act; NHS: National Health Service

Recommendation	Proposed actions	Existing initiatives
Strengthening AI governance in healthcare	Develop AI-specific regulatory frameworks (e.g., similar to clinical trial regulations for drugs) [12]	EU AI Act: classifies medical AI as "high-risk," requiring transparency and risk management [51]
	Classify high-risk AI systems (as per EU AI Act) and enforce stringent monitoring, transparency, and human oversight [51]	
	Expand FDA approval process for adaptive/self-learning AI [57]	FDA SaMD framework: regulates AI-driven medical devices [57]
	Establish mandatory AI bias assessments and require periodic audits of healthcare AI tools [59]	
	Include AI ethics experts in IRBs and hospital ethics committees for AI-based clinical decision-making systems [60]	UK MHRA AI Airlock: regulatory "sandbox" for testing AI-powered medical devices to address the challenge of AIaMD [84]
	Establish algorithmic accountability requiring entities that deploy AI to monitor their performance and outcomes continuously and to be answerable for any issues [58]	
Enhancing security and ethical AI development	Implement privacy-preserving AI training methods, e.g., federated learning, homomorphic encryption, and differential privacy for AI model training, minimizing direct data access supported through research grants and perhaps even enforced as policy for AI research [2]	HHS Hospital Cybersecurity Guidelines (USA): includes AI security measures [85]
	Strengthen cybersecurity mandates/audits and drills: require regular penetration testing, data encryption, and cybersecurity drills as part of hospital licensing [61]	IBM AI Fairness 360 Toolkit: open-source bias detection and mitigation in AI models [87]
	Enforce mandatory AI bias audits to prevent discrimination and bias in AI-driven diagnostics and treatment plans	Google’s Federated Learning: AI model training without centralizing data [86]
	Establish ethically-driven participatory/interdisciplinary AI development frameworks, requiring input from clinicians, ethicists, patients, and AI engineers from early development phases [62,63]	
	There is also a role for innovation sandboxes or pilot programs where AI can be tested in controlled environments under regulatory oversight to identify issues before wide rollout [64,65]	AI Bill of Rights blueprint introduced in the US, which calls for safe and effective systems, algorithmic discrimination protections, data privacy, notice/explanation, and human alternatives where needed [66,88]
Ensuring global and national standards alignment	Develop global AI certification/accreditation standards for healthcare AI tools for harmonization of AI regulations across countries (similar to CE marking in Europe or FDA approval in the US) [57,68,69]	OECD AI principles: guides global AI regulation [67]
	Implement "adequacy decisions" for cross-border health data sharing, ensuring secure and privacy-compliant research collaboration [70-72]	WHO-ITU Benchmarking for AI in Health: Global AI safety standards [75]
	Standardize state and federal laws in federated countries (e.g., India’s DPDP Act and US HIPAA) to prevent inconsistencies in data privacy governance [70-72]	EU GDPR Adequacy Decisions: Enables compliant data transfers between countries [76,77]
	Establish global frameworks for genomic AI ethics and regulate AI-driven precision medicine models [73,74]	International Declaration on Human Genetic Data (UNESCO): Framework for genomic data protection [89]
Education, training, and ethical culture	Integrate comprehensive AI literacy training, e.g., ethics, data stewardship, cybersecurity hygiene, and bias mitigation training into medical school curricula, AI engineers, and patients through CME to develop an AI culture in healthcare [78,79]	AMA AI in Medicine Course (USA): AI training for physicians [90]
	Establish "AI ethics committees" in hospitals and medical research institutions, overseeing algorithm review, clinical integration, and bias impact assessments [80-82]	NHS AI Ethics Advisory Group (UK): AI decision oversight in hospitals [91]
	Create patient advisory councils to provide real-world feedback on AI-based healthcare applications and data usage policies	
	Institutions should create an environment where raising concerns about an AI system’s behavior is welcomed and addressed	MIT AI Ethics Lab: training AI engineers in medical ethics [92]
	Develop interdisciplinary AI ethics workshops where data scientists, AI engineers, and clinicians collaborate on ethical AI use cases [78,79]	
Continuous policy iteration and research	Governments and professional bodies should regularly update AI policies/laws/regulations to address new challenges like generative AI in healthcare (e.g., chatbots, AI-generated medical summaries) [83]	India’s DISHA Bill: proposed framework granting patients greater control over health data [22]
	Introduce market-based incentives, encouraging private insurers and government payors to prioritize AI tools that meet fairness, privacy, and security benchmarks [83]	Germany’s DiGA Digital Health Applications: Insurance reimbursement for AI-driven medical tools [94]
	Promote multistakeholder AI governance models, incorporating perspectives from government agencies, healthcare professionals, tech companies, legal experts, and patient advocacy groups [83]	UK NHS Data Trust Model: Pilot initiative for ethical AI data governance [93]
	Establish new governance laws such as "data trusts," where patient data are managed by independent fiduciary entities for ethical AI research, ensuring compliance with privacy laws and patient rights [83]	

## Conclusions

AI is rapidly transforming healthcare delivery, but its benefits are inseparable from significant ethical, privacy, and governance challenges. Global regulatory frameworks such as GDPR, HIPAA, OECD, and WHO guidance converge on core principles of patient autonomy, data security, fairness, and accountability. Real-world case studies demonstrate that insufficient transparency, weak security safeguards, or biased algorithms can undermine public trust and expose institutions to legal and ethical risks.

India’s evolving legal framework, particularly the DPDP Act, represents an important step toward strengthening patient rights and accountability in digital health. However, effective implementation will require institutional capacity building, sector-specific guidance, and sustained oversight to translate statutory protections into everyday clinical practice. Across jurisdictions, key priorities include mitigating algorithmic bias, embedding privacy- and security-by-design in AI systems, ensuring meaningful informed consent, and maintaining clear human oversight of AI-supported decisions.

Ultimately, ethical AI in healthcare depends on governance models that integrate regulatory compliance with interdisciplinary collaboration and patient engagement. By aligning technological innovation with robust ethical and legal safeguards, healthcare systems can harness AI’s potential while preserving patient trust and protecting fundamental rights.
